# CoFactor: Folate Requirement for Optimization of 5-Fluouracil Activity in Anticancer Chemotherapy

**DOI:** 10.1155/2010/934359

**Published:** 2010-12-16

**Authors:** Muhammad Wasif Saif, Nektaria Makrilia, Kostas Syrigos

**Affiliations:** ^1^Clinical Medicine, The GI Oncology Section of the Division of Hematology/Oncology, Herbert Irving Comprehensive Cancer Center, Columbia University College of Physicians and Surgeons, Milstein Hospital, 177 Fort Washington Avenue, Suite 6-435, NY 10032, USA; ^2^Oncology Unit, Third Department of Medicine, Sotiria General Hospital, Athens Medical School, Mesogion 152, Athens 11527, Greece

## Abstract

Intracellular reduced folate exists as a “pool” of more than 6 interconvertable forms. One of these forms, 5,10 methylenetetrahydrofolic acid (CH_2_THF), is the key one-carbon donor and reduced folate substrate for thymidylate synthase (TS). This pathway has been an important target for chemotherapy as it provides one of the necessary nucleotide substrates for DNA synthesis. The fluoropyrimidine 5-fluorouracil (5-FU) exerts its main cytotoxic activity through TS inhibition. Leucovorin (5-formyltetrahydrofolate; LV) has been used to increase the intracellular reduced folate pools and enhance TS inhibition. However, it must be metabolized within the cell through multiple intracellular enzymatic steps to form CH2THF. CoFactor (USAN fotrexorin calcium, (*dl*)-5,10,-methylenepteroyl-monoglutamate calcium salt) is a reduced folate that potentiates 5-FU cytotoxicity. According to early clinical trials, when 5-FU is modulated by CoFactor instead of LV, there is greater anti-tumor activity and less toxicity. This review presents the emerging role of CoFactor in colorectal and nongastrointestinal malignancies.

## 1. Rationale

### 1.1. Folate Metabolism

Intracellular reduced folate exists as a “pool” of more than 6 interconvertable forms. One of these forms, 5,10 methylenetetrahydrofolic acid (CH_2_THF), is the key one-carbon donor and reduced folate substrate for thymidylate synthase (TS), the enzyme that catalyzes the methylation of deoxyuridine-5′-monophosphate (dUMP) to deoxythymidine-5′-monophosphate (dTMP). This enzymatic pathway provides one of the necessary nucleotide substrates for DNA synthesis. TS has, therefore, been an important target for cancer chemotherapy [1, 2].

The fluoropyrimidine 5-fluorouracil (5-FU) exerts its cytotoxic activity, at least in part, through TS inhibition. Cytotoxicity is achieved with the formation of 5-FU metabolite, a ternary complex consisting of fluorodeoxyuridine monophosphate (FdUMP), TS, and the reduced folate CH_2_THF [3–5]. However, since TS displays a Bi Bi kinetic mechanism of substrate recognition and binding, the presence of CH_2_THF as a TS cosubstrate is mandatory even in the absence of 5-FU (where no antineoplastic action is expected) [6–8].

Historically, leucovorin (5-formyltetrahydrofolate; LV) has been used to increase the intracellular reduced folate pools and enhance TS enzyme inhibition [7]. However, LV must be metabolized within the cell through multiple intracellular enzymatic steps to form CH_2_THF [9–12]. In general, CH_2_THF levels are relatively low, in the range of approximately 0.1–0.5 *μ*mol/L in normal cells, and even lower in human cancer biopsy tissues. While treatment with LV results in an approximately 2-fold increase in CH_2_THF [13, 14], maximum ternary complex formation is generally achieved at CH_2_THF concentrations approaching 12 *μ*mol/L [13].

Individual tumor metabolism of LV to tetrahydrofolate and, ultimately, to CH_2_THF is unpredictable, and CH_2_THF levels are typically among the lowest of the activated intracellular reduced folate forms [15, 16]. However, it is clear that high intratumoral levels of CH_2_THF allow for greater TS inhibition [17]. Preclinical and clinical investigations have consistently shown that resistance of tumors to 5-FU is associated, at least in part, with decreased intratumoral reduced folate levels, typically through decreased folylpolyglutamylation [17–20].

Thus, direct administration of the essential reduced folate CH_2_THF in place of LV might offer significant advantages with respect to clinical activity. This is supported by the fact that the essential reduced folate CH_2_THF is the direct cosubstrate of TS. LV, on the other hand, is a precursor and its bioavailability is partially exploited by other enzymes as well, since it is also the one carbon donor for other enzymatic pathways and provides several necessary substrates for DNA synthesis. This may also be linked to the fact that at CH_2_THF (monoglutamate) concentrations of above 1.0 microM, dUMP interference is nearly abolished, as folate levels influence the competitive basis and specificity of dUMP-mediated changes in the ternary complex formation [5].

### 1.2. CoFactor

CoFactor (USAN fotrexorin calcium, also known as *(dl*)-5,10,-methylenepteroyl-monoglutamate calcium salt and trivially as racemic CH_2_THF) is a reduced folate. In this protocol, CH_2_THF refers to the drug substance, and CoFactor refers to the clinical formulation. The drug is supplied as 100 mg of lyophilized powder in 10 mL vials. The lyophilized powder is reconstituted immediately before use with 10 mL sterile water for injection [21]. It should be pointed out that CH_2_THF acts as a cosubstrate, rather than a true cofactor, since it does not bound to the apoenzyme in order to form a holoenzyme, but instead must be present in stoichiometric concentrations to allow the conversion of an adequate amount of dUMP to its product. In order that the physiologic reaction as well as 5-FU action takes place, CH_2_THF must be present in conspicuous *in situ* concentrations within the cells.

## 2. Preclinical Studies

CoFactor has been shown to potentiate 5-FU cytotoxicity in numerous in vivo animal models of colorectal, pancreatic, and gastric cancers. Comparative analyses in “tumor take” models have demonstrated that CoFactor combined with 5-FU is significantly more effective in terms of synergistic antitumor activity when compared to LV/5-FU [22].

The combination of CoFactor and 5-FU/bevacizumab was also studied in an in vivo model using the HT-29 xenograft. CoFactor and LV significantly enhanced tumor inhibition and animal survival when added to 5-FU/bevacizumab, with the CoFactor arm appearing slightly better, albeit nonsignificant, than the LV arm [24]. A similar study using the DLD-1 human colorectal cancer xenograft model was conducted with the combination of 5-FU/oxaliplatin alone or in combination with LV or CoFactor. The CoFactor triple combination was clearly superior in inhibiting tumor growth, which translated in improved animal survival [23]. Regimens, protocols, and results of the preclinical studies have been summarized in Table 1.

## 3. Clinical Studies

### 3.1. Phase I/II Study in Patients with Advanced Cancers

In a phase I/II trial in patients with solid tumors, CoFactor was administered as intravenous (I.V.) bolus over 2–3 minutes at 2 dose levels, 100 mg or 200 mg, followed 20 minutes later by an I.V. bolus of 5-FU. The treatment was administered weekly [25].

Toxicities were mild, with only 2 of 17 subjects showing grade 3/4 gastrointestinal toxicity at the highest 5-FU dose (450–500 mg/m^2^) with the 200 mg dose of CH_2_THF. Mild conjunctivitis, however, was seen in a majority of subjects at that level, and grade 1/2 leucopenia was observed in only 6 of the 17 subjects.

Serial liver tumor biopsies obtained percutaneously in 17 patients after administration of the first dose of 5-FU/CoFactor confirmed potent inhibition of TS. Antitumor activity was observed: of 58 patients evaluable for tumor response, 17 exhibited a partial or complete response (29%). Clinical activity was principally seen in colorectal (*n* = 35; 33 evaluable, 7 responses), breast (*n* = 9; 5 responses), pancreatic (*n* = 5; 2 responses), and gastric (*n* = 9; 3 responses) cancers. No activity was seen in gallbladder cancer (*n* = 3; 2 evaluable, 0 responses). The median time to progression (TTP) for all patients was 265 days (range: 21–2221 days). In patients receiving 100 mg CoFactor/5-FU, the median time to progression was 443 days (range: 90–2221 days), which was significantly longer compared to 238 days (range: 90–1149 days) for patients receiving 200 mg CoFactor/5-FU (*P* = .0264).

Based on the report of reduced toxicity, the apparent greater activity reflected in TTP, and the adequate degree of inhibition of tumor TS activity, the dose of 60 mg/m^2^ (approximating the 100-mg dose) was selected as the phase II dose for CoFactor in combination with 5-FU [25].

### 3.2. Phase II Trial in Previously Untreated Metastatic Colorectal Cancer

A single-arm phase II clinical trial was conducted in 50 patients with previously untreated metastatic colorectal adenocarcinoma [26]. Dosing of CoFactor was based on the lower dose in the phase I study and administered as 60-mg/m^2^ I.V. bolus 20 minutes before 5-FU administration. 5-FU was administered at a dose of 450 mg/m^2^ bolus weekly for 6 weeks of a 7-week treatment cycle. Clinical response was evaluated after completion of 2 consecutive cycles and was based on objective tumor response (WHO criteria). The objective response rate (complete response plus partial response) was 35% (16 of 46 patients; 95% confidence interval, 21.4%–50.2%) as assessed by blinded third-party review. Median TTP was 162 days (approximately 5.3 months) from the beginning of treatment. Median overall survival was 459 days (approximately 15.1 months) as estimated by Kaplan-Meier projections.

The combination of CoFactor and 5-FU was well tolerated, and the safety profile was quite manageable. No cases of drug-related grade 3/4 gastrointestinal toxicity were observed. The incidence of myelosuppression was low, and granulocyte nadirs were only mildly decreased from baseline at the end of each treatment cycle. The highest individual grade of neutropenia was grade 2, which occurred in only one patient, and only one episode of grade 1 thrombocytopenia was recorded. Overall, this study suggested that this combination was associated with an improved safety profile when compared to 5-FU/LV [26]. 

### 3.3. Treatment after 5-Fluorouracil/CoFactor

Fifty patients completed CoFactor plus 5-FU treatment in the phase II clinical trial and continued with second-line therapy [27]. Four underwent partial liver resection for potential cure, and 29 patients received chemotherapy with irinotecan or oxaliplatin alone or in combination with 5-FU/LV as well as other agents. Seventeen patients received no poststudy intervention. Out of a total of 29 patients who received poststudy chemotherapy, 4 (14%) exhibited objective response, including one patient with complete response. Median overall survival, measured from the beginning of first-line treatment, was 23 months for patients who received second-line treatment, including patients who underwent surgical resection.

The combination of 5-FU and Cofactor has also been used as neoadjuvant chemotherapy prior to the resection of hepatic metastasis in colon cancer in an effort to reduce the necessary doses of 5-FU and related side effects [28]. The usefulness of such a combination in elderly patients should be explored further in future studies.

### 3.4. Randomized Controlled Phase IIB Trial with Previously Untreated Metastatic Colorectal Cancer (03-CoFactor Trial)

A recently completed randomized phase IIB study (study 03) compares CoFactor with LV, combined with infusional 5-FU, in 300 patients with previously untreated metastatic CRC. The primary endpoint of this study is the incidence of severe toxicity. The secondary endpoint of antitumor efficacy will be quality assured by an independent radiologic review. After the initial 150 patients had been enrolled onto the study, the Drug Safety Monitoring Board reviewed all of the interim data and recommended that the study continue as planned and final results are expected [29].

### 3.5. Randomized Phase III Trial in Metastatic Colorectal Carcinoma

This phase III study investigated the safety and efficacy of CoFactor in combination with 5-FU and bevacizumab versus LV plus 5-FU plus bevacizumab in metastatic colorectal carcinoma [30]. This was the first clinical trial that combined CoFactor and bevacizumab. The interim analysis showed that overall safety in patients receiving bolus Cofactor/5-FU plus bevacizumab was comparable to that in patients treated with LV/5-FU plus bevacizumab, constituting it a useful alternative. Results on progression-free survival and overall response rates are expected upon completion of the study [31].

### 3.6. Phase II Trials of CoFactor in Patients with Other Malignancies

The promising results of the initial studies that combined CoFactor and 5-FU in metastatic colorectal cancer led to the development of trials concerning the use of CoFactor in other malignancies as well. The ongoing Stewart et al. trial is a single-arm phase II study assessing the efficacy and safety of weekly bolus infusions in advanced breast cancer patients who failed prior treatment with anthracyclines and taxanes. Preliminary data on 21 patients showed that the CoFactor/5-FU combination is a highly effective, safe, and very well-tolerated treatment in metastatic breast cancer. With the use of anthracycline and taxanes-based regimens in the adjuvant setting, this combination could be a good treatment option in advanced disease [32]. A phase I/II clinical trial conducted in Europe studied the use of CoFactor in pancreatic cancer and demonstrated that CoFactor combined with 5-FU showed clinical benefit, defined as stable disease or tumor response, in 40% of patients. A phase III trial was planned but has not been performed yet [33].

### 3.7. Side Effects

Regarding adverse events reported with the administration of CoFactor/5-FU, the most frequent events are diarrhea, nausea, fatigue, and vomiting, but these are comparable to the adverse events noticed with LV/5-FU regimens [30–32]. Furthermore, it is not possible to distinguish whether these side effects derive from the CoFactor itself or the chemotherapy coadministered. After searching the published literature, we found no reports on possible drug interactions with other drugs aside from those with 5-FU that have been described above.

It should be noted that CH_2_THF cannot by itself be considered “toxic” unless referring to overdosage and/or side effects not related to the coadministration of 5-FU. They exert an ancillary action with 5-FU allowing TS inhibition to reach full completion, as it is needed for this reaction [5]. However, since folates have a major role in cell growth by serving as one-carbon unit donors for dTMP and purine ring biosynthesis, it can be argued that folate supplementation may fuel tumor growth, even in preclinical stages of malignancy. Therefore, folate administration must be performed with caution, and large trials are needed to clarify this subject.

## 4. Conclusion

As compared to LV, CoFactor exhibits a similar mechanism of action but is associated with reduced metabolic complexity, greater 5-FU antitumor activity, and more limited systemic 5-FU toxicity. It is a necessary cosubstrate for TS, which needs its CH_2_THF cosubstrate for 5-FU to gain full activity. It has been, therefore, proposed that CoFactor could be used instead of LV in current chemotherapy regimens containing 5-FU. CoFactor is not toxic per se and can be used when 5-FU is administered as a necessary part of the therapeutic protocol. The clinical efficacy in combination with the low toxicity of the 5-FU/CoFactor regimen suggests it might be a preferable initial treatment for metastatic colorectal cancer, especially in patients who require a less aggressive treatment. The latter may also enhance patient compliance. Preliminary data of ongoing studies show promising results of CoFactor in other malignancies as well. More large-scale randomized trials are needed to fully explore the potential of this novel agent.

##  Conflict of Interests

The authors declare no conflict of interests.

## Figures and Tables

**Table 1:** Preclinical studies of CoFactor.

Preclinical study	Study conducted on	Regimen	Protocol	Results
Bjelogrlić et al. (2007) [22]	human colon cancer cells: LS-174 and HT-29	CoFactor, LV, CoFactor/5-FU, LV/5-FU	Single agent concentrations ranged from 0.1 to 300 microM for 5-FU, CoFactor, and LV. In combined treatment, 5-FU concentration was matched with CoFactor or LV. Sulforodamine B cytotoxic test was used.	CoFactor showed cytotoxic effect on both cell lines. Addition of LV did not change 5-FU cytotoxicity, whereas, the combination of 5-FU with Co- Factor revealed synergistic and add- itive interactions.
Cantwell and Robbins (2005) [23]	athymic nude mice	LV- or CoFactor- regimens of 5-FU combined with: irinotecan, oxaliplatin, bevacizumab or gemcitabine	A human tumor xenotransplant model for colorectaland pancreatic cancer in athymic nude mice was used as well as an *in vivo* Balb/c systemic toxicity model.	CoFactor increases the therapeutic index of 5-FU- regimens since it induces equivalent or better antitumor response, less systemic toxicity and less weight loss as compared to LV-containing regimens.
Cantwell et al. (2004) [24]	6–8 week old nude mice inoculated subcutaneously with 2 × 106 HT-29 cells.	Combinations of 5-FU, CoFactor, leucovorin, and *α*VEGF (recomb- inant antibody, angiogenesis inhibitor)	When tumors reached 0.1–0.3 cm^3^ in volume, drugs were administered intraperitoneally. All drugs were dosed daily for 5 consecutive days with the exception of *α*VEGF, dosed on day 1. CoFactor or LV were injected 20 minutes prior to 5-FU.	Mean tumor volumes after Co- Factor/VEGF/5-FU combination we- re smaller than after 5-FU alone, CoFactor/FU, or leucovorin/5-FU. There was greater survival of mice treated with CoFactor/5-FU either with or without *α*VEGF compared to mice treated with only 5-FU.
